# An electronic registry to improve adherence to active surveillance monitoring among men with prostate cancer at a safety-net hospital: protocol for a pilot study

**DOI:** 10.1186/s40814-019-0482-x

**Published:** 2019-08-14

**Authors:** Benjamin Cedars, Sarah Lisker, Hala T. Borno, Puneet Kamal, Benjamin Breyer, Urmimala Sarkar

**Affiliations:** 1 Department of Urology, School of Medicine, University of California San Diego, 200 West Arbor Drive, San Diego, CA 92103 USA; 2 Center for Vulnerable Populations, University of California San Francisco, 2789 25th Street, San Francisco, CA 94110 USA; 3 Department of Medicine, School of Medicine, University of California San Francisco, 505 Parnassus Avenue, San Francisco, CA 94143 USA; 4 Department of Urology, School of Medicine, University of California San Francisco, 1001 Potrero Avenue, San Francisco, CA 94110 USA; 5 Department of Epidemiology and Biostatistics, School of Medicine, University of California San Francisco, 550 16th Street, San Francisco, CA 94158 USA

**Keywords:** Health information technology (HIT), Active surveillance, Prostate cancer, Systems Engineering Initiative for Patient Safety (SEIPS), Safety net, Patient safety

## Abstract

**Background:**

The evidence-based practice of active surveillance to monitor men with favorable-risk prostate cancer in lieu of initial definitive treatment is becoming more common. However, there are barriers to effective implementation, particularly in low-resource settings. Our goal is to assess the efficacy and feasibility of a health information technology registry for men on active surveillance at a safety-net hospital to ensure patients receive guideline-recommended care.

**Methods:**

We developed an electronic registry for urology clinic staff to monitor men on active surveillance. The health information technology tool was developed using the Systems Engineering Initiative for Patient Safety model and iteratively tailored to the needs of the clinic by engaging providers in a co-design process. We will enroll all men at Zuckerberg San Francisco General Hospital and Trauma Center who choose active surveillance as a treatment strategy. The primary outcomes to be assessed during this non-randomized, pragmatic evaluation are number of days delayed beyond recommended date of follow-up testing, the proportion of men who are lost to follow-up, the cancer stage at active treatment, and the feasibility and acceptability of the clinic-wide intervention with clinic staff. Secondary outcomes include appointment adherence within 30 days of the scheduled date.

**Discussion:**

Use of a customized electronic approach for monitoring men on active surveillance could improve patient outcomes. It may help reduce the number of men lost to follow-up and improve adherence to timely follow-up testing. Evaluating the adoption and efficacy of a customized registry in a safety-net setting may also demonstrate feasibility for implementation in diverse clinical contexts.

**Trial registration:**

ClinicalTrials.gov identifier NCT03553732, An Electronic Registry to Improve Adherence to Active Surveillance Monitoring at a Safety-net Hospital. Registered 11 June 2018.

## Background

Active surveillance (AS) is an increasingly acceptable strategy for treating patients with low- or intermediate-risk prostate cancer [1]. This management strategy is recommended for men who are likely to experience better or similar outcomes with careful monitoring and repeated testing than they would with active treatment, such as radical prostatectomy or radiation therapy [2]. It begins with shared decision-making between patients and physicians before screening, as well as coordination across care teams. Once selected, AS entails longitudinally following men with serial blood laboratory testing of prostate-specific antigen (PSA) levels and prostate tissue biopsies to monitor for disease progression. If disease progression occurs, patients can transition to active treatment.

However, despite evidence-based recommendations for AS in the right patient population and increased adoption as a management strategy, men are still not receiving timely and consistent monitoring [3, 4]. The vast majority of men on AS in clinical practice do not receive adequate monitoring according to the National Comprehensive Cancer Network (NCCN) guidelines for monitoring with PSA testing and prostate biopsy, and even fewer meet the more rigorous standards of clinical trial protocols [5, 6]. In a study by Luckenbaugh and colleagues, biopsy follow-up was discordant in 54% of men during the first 2 years of AS monitoring [6]. In another study examining follow-up beyond 2 years, the number of men who received biopsy declined to <13% [5]. Clinical trials indicate that a significant proportion of men on AS may develop more aggressive cancer [7]. Therefore, it is critical that they undergo monitoring so that progression to more aggressive cancer can be identified and treated in a timely fashion.

Scrupulous monitoring is even more critical at safety-net hospitals. Low-income patients and racial and ethnic minorities are more likely to seek care in safety-net health care settings where limited resources, including fragmented health information technology (HIT), and patient characteristics can introduce additional risks [8, 9]. Although some studies report that African American/Black men receive the same or slightly fewer follow-up PSA tests or prostate biopsies than Caucasian men, African Americans/Blacks are more likely to experience reclassification during surveillance and subsequently receive treatment for their prostate cancer [10, 11]. Krupski et al. demonstrated that low socioeconomic status was associated with increased uptake of conservative management [12]. It is unclear why this population of men receives conservative management more frequently. Active treatment may be more costly for uninsured populations, but in this care setting, cost differences are unlikely to play a role. Prior work suggests that men of lower socioeconomic status may be less likely to opt for surgery because of lack of trust [13]. In another study, loss to follow-up (LTFU) in AS was significantly higher at a safety-net hospital (57% at 5 years) compared to a university cancer center (37% at 5 years), and low socioeconomic status increased likelihood of LTFU [14]. Taken together, these findings suggest that safety-net hospitals serve patients who are more likely to select AS and also experience LTFU. These factors increase the risk of undetected progression of prostate cancer and resultant poor treatment outcomes. Vulnerabilities in the safety-net population, including mental health issues, non-English language, homelessness, substance use, and impaired literacy and numeracy, may contribute to sub-optimal adherence to AS. Therefore, it is imperative that we adopt strategies that increase the likelihood of a successful monitoring practice.

We plan to implement an AS monitoring intervention in the San Francisco Health Network, the city-funded integrated health care delivery system. The network is served by a single urology clinic located at Zuckerberg San Francisco General Hospital (ZSFG) and staffed by faculty and trainee physicians from the University of California, San Francisco. ZSFG has prior data of monitoring on AS from the Osterberg et al. study for comparison with the intervention [15]. The authors previously reported that 18.3% of the men on AS at this facility exhibited cancer upgrade and 17% were LTFU. The current system in place at the urology clinic utilizes an Excel spreadsheet to monitor patients on AS. We aim to perform a pilot study implementing a novel, co-designed HIT platform in an effort to improve the monitoring of and adherence to the recommended AS guidelines. The pilot study will test the feasibility, acceptability, and preliminary estimates of efficacy in utilizing a HIT tool for AS in the urology clinic at ZSFG. This pilot is necessary to determine whether the effort to systematize monitoring for AS improves patient outcomes. Based on the initial results of primary and secondary outcomes, we will iterate the platform for use in the ongoing study.

## Methods

### Intervention development

To inform this intervention, we used the Systems Engineering Initiative for Patient Safety (SEIPS) model that has previously been applied in an outpatient surgery context [16]. SEIPS targets three basic interconnected elements of a clinic—work system (or organizational structure), process, and outcome—for potential intervention. We utilized a human factors design approach frequently used across industries, called journey mapping, to elicit details from doctors, nurses, nurse practitioners, and other stakeholders involved in the monitoring process about a patient’s journey through the outpatient urology clinic while on active surveillance [17–20]. Journey maps allow for identification of steps in the monitoring process that are particularly vulnerable to gaps in patient safety. From the journey map, we identified vulnerabilities in patient safety domains including tasks, technology, organization, people, and environment [21, 22]. Vulnerabilities included patient challenges (homelessness, substance abuse, and mental illness), the cognitive load of tracking patients, time-limiting factors such as the rotating schedules of residents, and overall task burden on providers.

In order to develop a viable patient monitoring system, we used a novel design seed method to address these vulnerabilities [17, 23, 24]. Design seeds take the place of the typical technical approach that moves from problem to solution by serving as a bridge between vulnerabilities experienced and solution attributes. They serve as modular and evaluable “seeds” to solutions that promote early and iterative evaluations before investing in a full-fledged solution. This process was refined through multiple iterations with input from providers to produce a finely tuned tool specific to the needs of the urology clinic.

### Health information technology tool

**Fig. 1:**
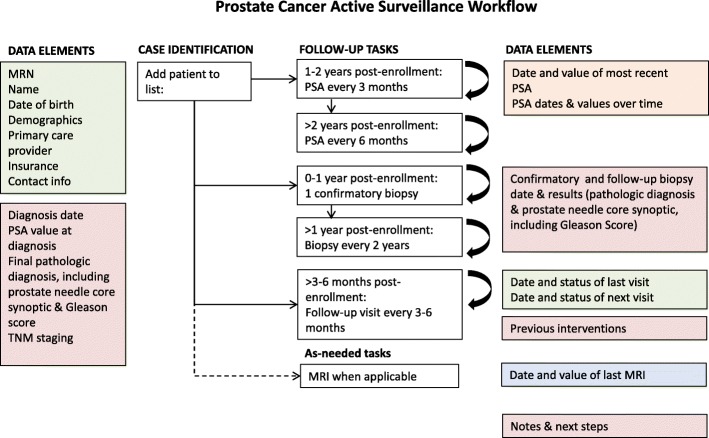
Workflow diagram depicting the various elements of the HIT tool

**Fig. 2:**
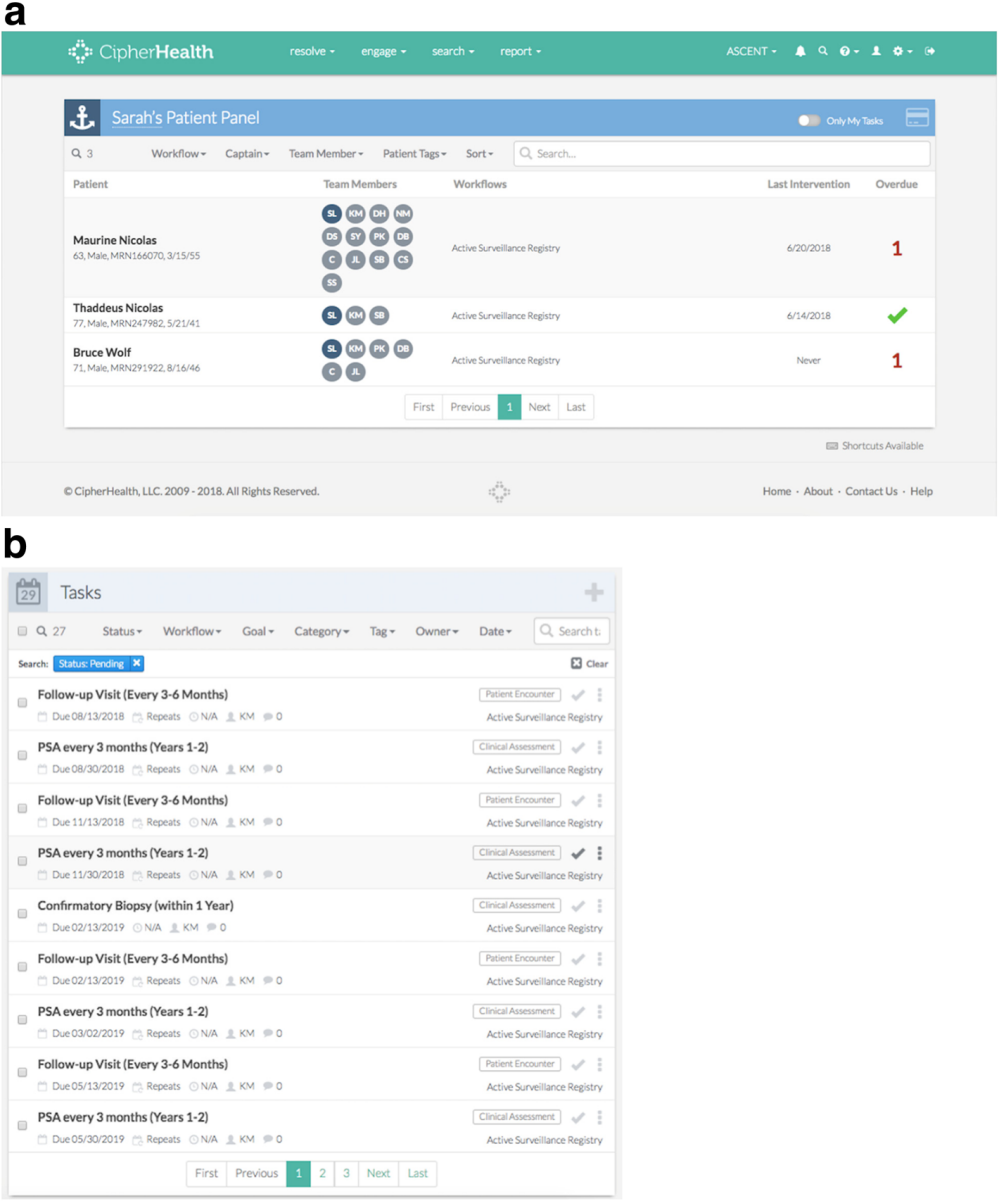
**a** Screenshot depicting the population-level view of patients on AS. The patient names are fictitious for demonstration purposes. **b** Screenshot of AS follow-up tasks on the patient level

The electronic monitoring tool was customized for use in the urology outpatient clinic for men on AS. The top five design seeds identified in the urology clinic as most important for improving monitoring and saving time were as follows: “keeps list up to date,” “customize the patient list,” “ability to control data access,” “population registry functionality for high-risk patients,” and “assign roles and responsibilities” [17]. Based on these results and other input from medical professionals with knowledge of the unique challenges facing the clinic, we partnered with CipherHealth (New York, NY), a healthcare technology company, to develop a registry to aid in patient monitoring. This tool consolidates patient information from three major data sources and provides clinicians with the ability to track patients on AS to ensure up-to-date and timely care (Fig. 1). Aside from the automatic feed of data from Openlink, manual data entry by clinical staff is also possible. The AS registry tracks and notifies the clinic team when testing is due, according to the recommended AS protocol (Fig. 2a) [25–27]. This includes PSA testing every 3 months for the first 1–2 years post-enrollment, PSA every 6 months > 2 years post-enrollment, 1 confirmatory biopsy within 12 months of enrollment, biopsy every 2 years > 1 year post-enrollment, follow-up visit every 3–6 months post-enrollment, and MRI as needed (Fig. 2b).

### Study design

This study is a prospective non-randomized pilot study that will add newly diagnosed men with prostate cancer who choose AS as an initial management strategy to the registry and follow them. We will also continue to track men who are already on AS at ZSFG, adding them into the new system. Patients will be entered into the registry by involved healthcare providers. A team of care managers, including a nurse practitioner and medical residents, will perform registry enrollment and maintenance. As the team of medical residents rotates, the study group will conduct recurring training sessions to ensure all users are able to access and use the tool. In our case, this is approximately every 4 months. When the residents return to the urology clinic on rotation, they can engage in peer-training. The study team and CipherHealth provide ongoing support for the clinical staff. CipherHealth offers a data portal for the study team to be able to capture real-time metrics, such as number of patients enrolled, tasks completed, and outcomes. The registry will provide automated reminders to prompt follow-up activities (e.g., visits, testing, check-ins). Eligibility criteria for AS include diagnostic PSA ≤ 10 ng/ml, clinical stage T1 or T2, Gleason scores ≤ 3 + 4, ≤ 33% positive cores, and ≤ 50% tumor in any single core. The frequency and cadence of follow-up tests will be tracked and compared to predetermined recommended guidelines. We will monitor deviations from an established timeline including but not limited to delayed or missed PSA testing, delayed or missed prostate biopsy, and LTFU as defined by no PSA test or prostate biopsy for 18 months at ZSFG or a participating hospital in the health information exchange. We will also record definitive treatment, modality, and reason for changes in management, as well as patient-related outcomes such as overall and prostate cancer-specific mortality. There will be a minimum follow-up period of 2 years to make baseline inferences about the efficacy and adoption of the registry, with continuous accrual extending indefinitely. We will descriptively compare outcomes from this new cohort of AS patients to the results from the Osterberg et al. study, including proportion of men LTFU, average number of PSA measurements and prostate biopsies, and time from diagnosis to active treatment. At this juncture, with 2 years of data and an understanding of how well the registry has been integrated into clinical workflows, appropriate adjustments will be implemented for the continuation of the study.

### Patient population

The goal of the study is to enroll all patients in the clinic who are undergoing AS. There are currently 44 patients who have been added to the registry; 40 of whom are being managed by AS. Of the remaining four men, one began active treatment, one declined further treatment, and two have transferred care elsewhere. These patients have been on AS for a median of 3.2 years.

The prior AS study at the ZSFG demonstrated that patients at the urology clinic had median age at prostate cancer diagnosis of 61.5 years (range 44–81 years) [15]. The racial composition was 29% African American/Black, 25% White, 30% Asian/Pacific Islander, and 15% Hispanic/Latino. Sixty-four percent of men primarily spoke English, 9% Spanish, 16% Chinese, and 12% other. For all ambulatory surgery patients at ZSFG, more than two thirds are insured by Medi-Cal (California’s Medicaid Program) [28].

### Medical record review

Patients with confirmed pathologic diagnosis of prostate cancer seen at least once in the clinic will be eligible for record review. Data abstraction will include sociodemographic, clinical, and treatment variables, including age, race, PSA level at diagnosis, clinical stage, Gleason score, and treatment modality (Table 1).

**Table 1:** Domains for data abstraction from medical records of patients who enroll in AS

Domain	Data element
Demographics	Age
	Sex
	Race/ethnicity
	Primary language
	Insurance type
Social history	History of tobacco use
	History of substance abuse
	History of homelessness
	Employment history
Clinical characteristics	PSA at diagnosis
	Clinical T-stage
	Gleason score
	Positive biopsy cores
	Treatment modality (if discontinue AS)
Medical history	Number of comorbidities
	History of mental illness
	Family history of prostate cancer

### Outcomes

Primary outcomes include the number of days delayed past recommended follow-up interval (continuous variable), proportion of men who are LTFU, cancer stage at time of active treatment, and clinic team acceptability and feasibility of the registry tool. Secondary outcomes include appointment adherence within 30 days (binary) (Table 2). We will measure the feasibility and acceptability of the HIT tool among clinic staff using semi-structured interviews. We will interview at least one clerk responsible for scheduling, one registered nurse, one nurse practitioner, and at least three urologists. We will not be aiming for a specific proportion of staff, but rather representation of all types of staff. The interviews will be conducted once the registry has been in use for 6 months. If staff who have been involved with the system leave the team, we will conduct exit interviews to ensure their voices are included. These interviews will be based on the principles of perceived usefulness and ease of use from the technology acceptance model framework [29]. Among other parameters, this framework investigates usefulness, ease of use, relevance, and result demonstrability. Using a grounded theory approach, we will analyze semi-structured interviews abductively, integrating inductive and deductive reasoning to explore and describe emergent themes within structured domains of interest. Within each domain, we will iteratively open-code, analyze, and theorize until we have reached saturation and no more themes emerge. Finally, we will see if we can integrate themes across domains for a unified theory [30]. Our definition of an effective intervention is one that limits the delay in receiving follow-up tests, reduces the number of men LTFU, and promotes active treatment at lower cancer stages (e.g., non-metastatic disease), and one that the clinical team rates highly in terms of feasibility and acceptability.

**Table 2:** Primary and secondary outcomes

Outcomes		Objectives
Primary outcomes	Number of days delayed past follow-up date	To improve adherence to AS protocol
	Proportion of men LTFU	To reduce LTFU
	Cancer stage at active treatment	To prevent progression by improved detection
	Acceptability and feasibility of the HIT tool	To improve uptake and sustained use of the tool in clinical practice
Secondary outcomes	Appointment adherence within 30 days	To improve adherence to AS protocol

### Analyses

Descriptive statistics will be used to report patient demographics, clinical characteristics, and treatment decisions, with medians and ranges or frequency and percentages depending on the type of data. Kaplan-Meier estimates will measure adherence to AS. Multivariate logistic regression will predict risk of non-adherence, definitive treatment, biopsy upgrade, and mortality. Covariates will include age, race, PSA at diagnosis, Gleason grade, clinical stage, and number of comorbidities.

## Discussion

Active surveillance for men with low-risk prostate cancer is a safe and effective management strategy. However, this process occurs over a time period of years and requires ongoing patient adherence to blood tests, appointments, and prostate biopsies, which are invasive medical procedures. This complex, long-term follow-up model presents challenges to adherence and proper delivery of care. The San Francisco Health Network is a publicly funded health system with circumscribed clinical personnel, fragmented electronic health records, and a diverse patient population that make AS monitoring particularly challenging. Furthermore, one study warned that AS may perform poorly at identifying African American/Black men with low-risk prostate cancer based on adverse pathology (i.e., worse cancer stage or Gleason grade) at radical prostatectomy [31]. This prospect makes thorough and timely follow-up essential due to the increased risk of under-staging.

The digital registry system we created will give providers the ability to track appointments, follow-up tests, and results. This system is independent from the general medical record and is therefore unencumbered by the heterogeneity of paper or various electronic medical record systems. The implications of a successful electronic monitoring system may reach beyond this single institution. A successful registry tool that can demonstrate better adherence to AS and less LTFU could serve as a template for other urology clinics in resource-constrained contexts. In turn, this has the potential to reduce disparities in prostate cancer outcomes, because patients at most risk for poor outcomes are disproportionately cared for in safety-net settings.

Potential challenges include technical difficulties with the tool itself, poor adoption of the tool by providers, or misuse, as well as unforeseen issues. However, the monitoring system was designed in a theory-informed manner and tailored to meet the needs and specifications of the clinic. We expect that the adapted design approach will enable accelerated adoption and efficacy in monitoring men with prostate cancer and thereby improve patient outcomes.

## Data Availability

Available from the corresponding author on reasonable request.

## Abbreviations

AS: Active surveillance
HIT: Health information technology
LTFU: Loss to follow-up
NCCN: National Comprehensive Cancer Network
PSA: Prostate-specific antigen
SEIPS: Systems Engineering Initiative for Patient Safety
ZSFG: Zuckerberg San Francisco General Hospital and Trauma Center
